# Combined use of topical intraarticular tranexamic acid and rivaroxaban in total knee arthroplasty safely reduces blood loss, transfusion rates, and wound complications without increasing the risk of thrombosis

**DOI:** 10.1186/s12891-018-2151-2

**Published:** 2018-07-18

**Authors:** Yong Tae Kim, Min Wook Kang, Joon Kyu Lee, Young Min Lee, Joong Il Kim

**Affiliations:** 1 Department of Orthopaedic Surgery, Hallym University Kangnam Sacred Heart Hospital, 1, Singil-ro, Yeongdeungpo-gu, Seoul, 150-950 South Korea; 2 Department of Orthopaedic Surgery, Hallym University Sacred Heart Hospital, 22, Gwanpyeong-ro 170beon-gil, Dongan-gu, Anyang, 431-796 South Korea

**Keywords:** Total knee replacement, Tranexamic acid, Rivaroxaban, Blood loss, Transfusion, Wound complication, Deep vein thrombosis

## Abstract

**Background:**

Blood loss and deep vein thrombosis (DVT) are important complications after total knee arthroplasty (TKA). Topical tranexamic acid (TXA) effectively reduces wound bleeding but may elevate the risk of DVT. In contrast, rivaroxaban potently prevents DVT but has been associated with bleeding complications. The simultaneous use of topical TXA and rivaroxaban in TKA has not been much investigated.

**Methods:**

A retrospective cohort study was conducted with two consecutive groups of patients who underwent TKA. Intraoperatively, one group (RVTX group) received topical, intraarticular TXA, while the other (RV group) did not. Both groups were administered rivaroxaban postoperatively for 14 days and underwent Doppler ultrasound for DVT screening. After propensity score matching, both groups consisted of 52 patients (104 patients in total) and were compared regarding total drain output, nadir haemoglobin (Hb), maximum Hb decrease, calculated total blood loss, transfusion rate, and incidence of DVT and wound complications.

**Results:**

Both groups showed no significant differences in the propensity-matched variables of age, sex, body mass index, American Society of Anesthesiologists physical status score, and preoperative Hb. The RVTX group showed a significantly higher nadir Hb (*p* < 0.001), lower drain output (*p* < 0.001), Hb decrease (*p* = 0.015), total blood loss (*p* < 0.001), and rate of transfusion (*p* < 0.001) and fewer wound complications (*p* = 0.027). However, the incidence of DVT (*p* = 1.000) did not differ significantly between the two groups, and all cases were asymptomatic.

**Conclusions:**

The combined use of intraarticular topical TXA with rivaroxaban in patients undergoing TKA is a safe and effective method to reduce blood loss, the need for transfusion, and wound complications without elevating the risk of DVT.

## Background

Total knee arthroplasty (TKA) involves a significant amount of blood loss due to extensive bone cuts and soft tissue dissection. Persistent bleeding may increase the risk of infection, worsen the postoperative wound condition, cause transfusion-related complications such as immunologic rejection and disease transmission, and increase costs [1–4]. Among the various options in addition to standard tourniquet use, the antifibrinolytic agent tranexamic acid (TXA) has shown promising results due to its ease of use, relatively low cost, and high haemostatic potency [5–7]. However, concerns exist regarding its possible systemic effect on elevating thrombotic risk, especially when no or a weak concurrent thromboprophylaxis regimen is used [8]. Thus, topical application of TXA has been investigated and found to be comparable to intravenous injection for decreasing transfusion rates [9].

Deep vein thrombosis (DVT) is of a major concern in TKA because it is the main cause of postoperative pulmonary embolism, a potentially life-threatening complication. The natural incidence of DVT after TKA without prophylaxis is reported to be as high as 45–56% [10, 11]. Therefore, thromboprophylaxis, traditionally using low molecular weight heparin (LMWH), warfarin, and aspirin, has long been a standard postoperative protocol. However, oral agents such as rivaroxaban, apixaban, and dabigatran have recently been introduced due to their advantages of easier administration, no need for monitoring, and increased or equivalent potency compared to classic anticoagulants. Among these oral drugs, rivaroxaban demonstrated the most effective protection against DVT [12]. However, studies have shown higher incidences of wound bleeding and deep surgical site infection [13, 14] in patient groups who were given rivaroxaban after TKA.

Though TXA and rivaroxaban are each highly effective for their approved purposes, the benefits may be overshadowed by higher risks of DVT and wound complications, respectively [8, 13, 14]. However, it is plausible that when TXA and rivaroxaban are used together, the actions of one drug may compensate for the adverse effects of the counterpart drug. Nevertheless, many studies regarding the use of TXA in TKA involved a thromboprophylaxis regimen other than rivaroxaban [15–19]; only a few analysed the concurrent use of TXA with rivaroxaban in TKA [20–22]. In most of these studies, TXA was administered intravenously, although topical TXA has been shown to be noninferior to intravenous injections in reducing blood loss, with a minimal resultant systemic concentration [19, 23]. Thus far, the concurrent use of topical TXA and rivaroxaban has not yet been investigated extensively.

The objective of this study is to evaluate the efficacy and safety of the combined regimen of topical TXA with rivaroxaban in TKA by comparing groups with or without topical TXA use, both with thromboprophylaxis via rivaroxaban. The main hypothesis was that the intraarticular injection of TXA after capsule closure, combined with rivaroxaban use for thromboprophylaxis, would result in a smaller postoperative haemoglobin (Hb) decrease, fewer transfusions and wound complications, and no increase in DVT risk.

## Methods

### Inclusion and exclusion criteria

After the approval by the Institutional Review Board of the authors’ institute (HUKSHH IRB 2017–10-010) and in accordance with the Declaration of Helsinki, a single-centre retrospective cohort study was performed on patients who (1) received primary unilateral TKA for degenerative arthritis, (2) received postoperative rivaroxaban for thromboprophylaxis, and (3) either received or did not receive topical TXA for haemostasis.

In February 2015, rivaroxaban was selected as the primary pharmacologic DVT prophylactic agent after TKA in the authors’ institute. Previously, LMWH was the first line treatment, and for patients who refused subcutaneous injection, aspirin was offered. Starting in March 2017, due to growing reports of postoperative bleeding, intraarticular injection of TXA after capsule closure was added to the routine surgical procedure. There was no bias in case selection when using TXA, as consecutive patients underwent the default perioperative protocol solely depending on the operation date.

The published contraindications [24, 25] for each drug were strictly obeyed; patients who could not receive rivaroxaban or topical TXA were not included in the study. Patients who could not meet the preoperative Hb requirements (> 10 g/dL) and required preoperative transfusion were excluded. Patients who received implants other than those specified in the latter section due to a previous contralateral implant of another design were excluded. Cases in which patellar resurfacing was impossible due to an overly thin or small patella were also excluded. Such exclusions were made to minimize the confounding factors and set the use of topical TXA as the only independent variable.

The total number of patients included was 106 for the rivaroxaban-only (RV) group, and 52 for the rivaroxaban plus topical TXA (RVTX) group.

### Surgical intervention

**Fig. 1:**
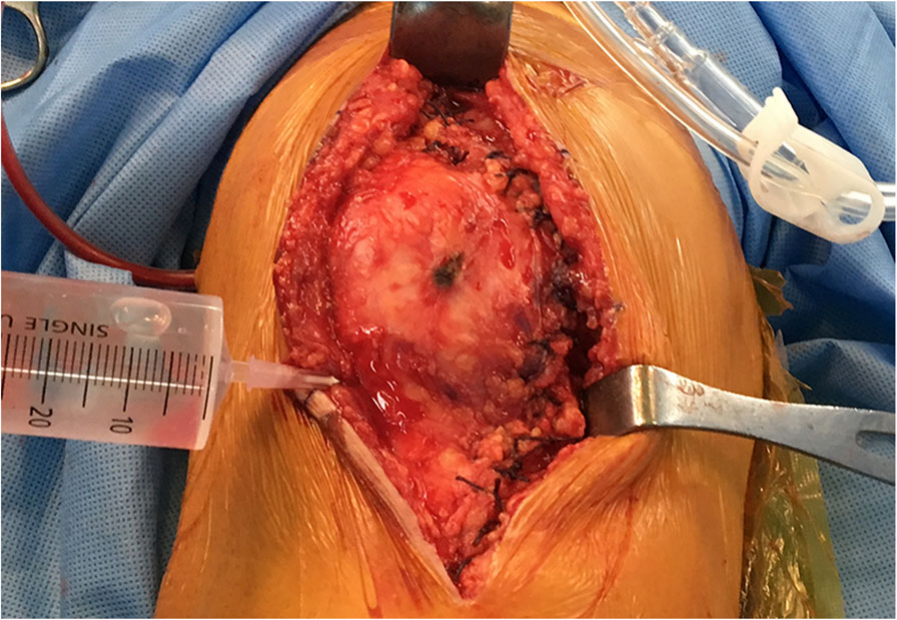
Intraarticular injection of tranexamic acid after capsule closure. After watertight capsule closure, a solution of 1 g tranexamic acid in 50 mL normal saline was injected intraarticularly into the knee joint. Leakage of the solution was further monitored through the range of motion. Note the clamped drainage tube

All operations were performed through a standard medial parapatellar approach under spinal anaesthesia. A pneumatic tourniquet was inflated to 300 mmHg immediately before incision. All patients received the same cemented posterior stabilized implant (Persona®, Zimmer, Warsaw, IN), and the patella was resurfaced. After final implant fixation and removal of excess cement, gauze packing was performed, and the tourniquet was deflated. Manual compression around the surgical field was applied until the cement had completely hardened, and bleeding foci evident after gauze removal were identified and cauterized. A clamped closed suction drain (Barovac®, Sewoon Medical, Cheonan, Korea) was placed inside the joint, and a watertight closure of the capsule was performed. At this point, the RVTX group received an intraarticular injection of 1 g of TXA (Tranexamsan®, Shinpoong Pharmaceutical, Seoul, Korea) mixed in 50 mL normal saline (Fig. 1). The solution was left in the joint with the drain clamped. After the injection, the knee was moved throughout the range of motion to confirm the watertight closure of the capsule. In both groups, the drain was unclamped after two hours and was removed 48 h after the surgery. No preoperative autologous blood transfusions or intraoperative blood salvage were performed.

### Postoperative management

Both groups shared the same postoperative protocol. Immediately after surgery, an intermittent pneumatic compression pump (Flowtron®, ArjoHuntleigh, Addison, IL) was applied, and gentle compressive dressing of the operated leg was maintained until drain removal at 48 h postoperatively. Then, the compressive dressing was changed to a light adhesive dressing, and continuous passive motion was initiated. Tolerable weight bearing and early mobilization were encouraged.

Postoperative fluid replacement included standardized daily administration of 500 mL of hydroxyethyl starch volume expander (Volulyte®, Fresenius Kabi, Bad Homburg, Germany) on postoperative days (POD) 1 and 2. Transfusion of allogeneic blood was indicated only when the Hb concentration decreased below 8 g/dL. Laboratory tests including Hb were performed on the day before surgery and on POD 1, 2, 4, and 6.

From POD 2 to 14, patients received a daily dose of 10 mg rivaroxaban (Xarelto®, Bayer HealthCare AG, Wuppertal, Germany). On POD 6, all patients, regardless of the presence of clinical symptoms of DVT, underwent a bilateral diagnostic Doppler ultrasound examination. If the findings were negative, patients were discharged on POD 7. Wound complications including wound dehiscence, hematoma, and surgical site infection were monitored during the hospital stay and after discharge until four weeks postoperatively at the outpatient clinic.

### Data collection

The following data were retrieved retrospectively from medical records: demographic data including age, sex, and body mass index (BMI); physical status-related parameters including preoperative Hb (in g/dL) and American Society of Anesthesiologists physical status classification (ASA-PS); outcome measures related to blood loss including total drain output (in mL), nadir Hb during the hospital stay, maximum decrease in Hb (calculated by the nadir Hb subtracted from the preoperative Hb), total blood loss (in mL, calculated by the formulas published by Nadler et al. [26] and Good et al. [7]), and postoperative records of transfusion; routine Doppler ultrasound results; and wound complications.

### Statistical analysis

To minimize possible confounding factors, both groups underwent propensity score matching prior to analysis. The variables matched included demographic data including age and sex and preoperative physical status data including BMI, ASA-PS, and preoperative Hb. The match tolerance, the maximum difference between propensity scores of any matched pair, was set to 0.1. As the number of patients in the RV group (*n* = 106) was approximately twice that in the RVTX group (*n* = 52), every patient in the RVTX group was matched to a patient in the RV group (the RV group hereafter refers to the group of 52 patients who were each matched with a patient of the RVTX group).

Further analysis was performed with an identical number (*n* = 52) of patients in both groups. For continuous data, independent t-tests were applied to express results as means and 95% confidence intervals (CI). Pearson chi-square and Fisher’s exact tests were used to compare percentages for binary data.

A *p*-value less than 0.05 was considered statistically significant. IBM SPSS Statistics for Windows, Version 24 (IBM Corp., Armonk, NY) was used for propensity score matching and subsequent statistical analyses.

## Results

The propensity matched variables of age, sex, BMI, preoperative Hb, and ASA-PS were not significantly different between the two groups (Table 1).

**Table 1:** Propensity score matched data

	Rivaroxaban only (*n* = 52)	Rivaroxaban + IA TXA (*n* = 52)	*p*-value
Age (years)^a^	70.4 (68.0–72.9)	72.0 (70.0–74.0)	0.320
Male:Female^b^	6: 46	8: 44	0.775
BMI (kg/m^2^)^a^	25.1 (24.2–26.1)	25.0 (24.4–25.7)	0.816
ASA-PS classification^a^	2.1 (1.9–2.2)	2.1 (2.0–2.3)	0.704
Preoperative Hb (g/dL)^a^	12.5 (12.1–12.9)	12.6 (12.2–13.0)	0.630

*IA TXA* Intraarticular tranexamic acid, *BMI* Body mass index, *ASA-PS* American Society of Anesthesiologists Physical Status, *Hb* Haemoglobin

^a^Values presented as means (95% confidence interval) and compared by independent t-tests

^b^Values presented as proportions and compared using Pearson’s chi-square test

However, the RVTX group demonstrated a significantly higher nadir Hb during the hospital stay (*p* < 0.001) and a lower total drain output (*p* < 0.001), maximum decrease in Hb (*p* = 0.015), and total blood loss (*p* < 0.001) (Table 2). The RVTX group also demonstrated a significantly lower rate of postoperative transfusion (*p* < 0.001) (Table 3).

**Table 2:** Continuous outcome measures

	Rivaroxaban only (*n* = 52)	Rivaroxaban + IA TXA (*n* = 52)	*p*-value
Total drain output (mL)	1021.4 (893.9–1148.9)	419.1 (349.9–488.3)	< 0.001
Nadir Hb during hospital stay (mL)	8.2 (7.9–8.6)	9.0 (8.8–9.3)	< 0.001
Maximum decrease in Hb (g/dL)	4.2 (3.9–4.6)	3.6 (3.1–4.0)	0.015
Total blood loss (mL)	1260.1 (1168.7–1351.6)	1008.2 (910.3–1106.2)	< 0.001

All values presented as means (95% confidence interval) and compared using independent t-tests

**Table 3:** Binary outcome measures

	Rivaroxaban only (*n* = 52)	Rivaroxaban + IA TXA (*n* = 52)	*p*-value
Transfusions^a^	26 (50.0%)	8 (15.4%)	< 0.001
Ultrasound-diagnosed DVT^b^	2 (3.8%)	3 (5.8%)	1.000
Wound complications^b^	6 (11.5%)	0 (0%)	0.027

All values presented as the number of patients (percentage)

^a^Compared using Pearson’s chi-square test

^b^Compared using Fisher’s exact test

The incidence of DVT was not significantly different between the two groups (Table 3). A total of five patients, two from the RV group and three from the RVTX group, were diagnosed with isolated distal DVT by routine ultrasound examinations. However, none of the patients complained of any clinical symptoms, and further studies also excluded pulmonary thromboembolism. All patients were treated uneventfully with an extended daily dose of 20 mg of rivaroxaban for three months. During follow-up, no wound complications occurred in these patients.

However, a significant difference was observed in the occurrence of wound complications (Table 3). Although no cases of wound dehiscence or surgical site infection occurred, six patients, all from the RV group, developed a subcutaneous hematoma. Three patients required wound revision including hematoma removal, while in the other three patients, the hematomas resolved spontaneously with ice application and gentle compression.

## Discussion

The present study showed that the combined regimen of topical TXA and rivaroxaban significantly reduces postoperative blood loss, the transfusion rate, and wound complications, without increasing the risk of DVT.

Several studies so far have investigated the combined use of TXA and rivaroxaban in TKA. With intravenous TXA, Wang et al. [17] reported less blood loss and a lower rate of transfusion and wound complications in the rivaroxaban-IV TXA group, with no significant difference in DVT occurrence. Wood et al. [20] also reported a significantly reduced need for transfusion in the same patient group. Similar results with intraarticular TXA were produced by Wang et al. [22] with a dose of 0.5 g in a 10 mL solution, and Yen et al. [27] with a dose of 3 g in a 100 mL solution.

The classic arsenal of chemical thromboprophylaxis has limitations such as the need for daily injections (LMWH, enoxaparin, fondaparinux), a narrow therapeutic window (warfarin), or insufficient protection (aspirin) [28, 29]. Therefore, surgeons are now focusing on novel agents with potency matching or exceeding the traditional options, ease of oral administration, and no need for constant monitoring [30]. These options include the direct factor Xa inhibitors rivaroxaban and apixaban, and the direct thrombin inhibitor dabigatran [25, 31, 32]. Gómez-Outes et al. [12] compared these three oral anticoagulants with enoxaparin and showed rivaroxaban to be the most effective agent.

However, rivaroxaban has been associated with complications related to bleeding due to its potent anticoagulant properties. Wang et al. [21] demonstrated that in patients given rivaroxaban alone after TKA, compared to patients with additional intravenous TXA, significantly higher incidences of ecchymoses and wound hematoma were recorded. Ricket et al. [14] compared patients who received either rivaroxaban or enoxaparin and described a higher rate of clinically relevant non-major bleeding, including surgical site bleeding and hematoma. In a study by Brimmo et al. [13], compared to patients who received thromboprophylaxis other than rivaroxaban, those who received rivaroxaban after lower extremity arthroplasty demonstrated a significantly higher rate of early deep surgical site infection. Although the present study did not report any case of infection, a significantly higher incidence of wound hematoma was observed in the RV group, confirming the previous findings in the literature. In the period during which rivaroxaban was used without TXA, the authors became aware of the increasing number of cases of wound bleeding and sought additional TXA administration.

Simultaneously achieving postoperative haemostasis and thromboprophylaxis is complex, as the respective drugs are expected to exert opposite effects of coagulation and anticoagulation. While chemical thromboprophylaxis has long been regarded as a part of the standard treatment, pharmacologic treatments of postoperative haemostasis are not as popular, mostly due to concerns that such treatments may contribute to thrombogenesis [33–35]. However, TXA is an antifibrinolytic agent and does not trigger a coagulation cascade, but instead exerts its haemostatic effect by inhibiting the degradation of fibrin already formed at the bleeding foci [36]. Therefore, the safety of using TXA in TKA, which resulted in no significant elevation in DVT risk, has been reported in the literature [15, 16, 37]. However, all these studies had some form of concurrent thromboprophylaxis, which may have masked the possible elevation of DVT risk due to TXA. Thus, Nishihara et al. [8] conducted a study of intravenous TXA use with only mechanical, but no chemical, thromboprophylaxis. The results showed that compared to the group not administered TXA, the intravenous TXA group showed a significantly higher incidence of isolated distal DVT.

Therefore, topical TXA was administered in the present study to reduce the effects of rivaroxaban at the surgical site while minimizing the possible systemic effects of TXA in elevating DVT risk. This decision was supported by Wong et al. [23], who showed that topical TXA, compared to intravenous injection, demonstrated a lower serum concentration while maintaining a maximum level at the surgical site. Regarding the efficacy of TXA, in a meta-analysis [9] of six studies comparing topical and intravenous TXA, topical TXA was similarly effective in decreasing blood loss after TKA. The present study also strengthened these findings, as patients treated with intraarticular injections of TXA showed significantly less blood loss and lower rates of transfusion, without a significant elevation in DVT risk.

Currently, the optimal protocol for topical TXA administration has not been established in the literature. Intraarticular injection with or without drain clamping, impregnation for a given time and removal of the residual solution within the open joint, or irrigation with the solution have been investigated as viable methods, and all showed significantly less blood loss than with no TXA use [9, 19, 38]. However, in the present study, routine intraarticular injection with the drain clamped offered a method of confirming watertight capsule closure, which may have been helpful in preventing subcutaneous hematomas and retrograde infections. Moreover, unlike impregnation, which requires a certain amount of time before removal of the solution, intraarticular injection required no delays but still provided a long contact time, maximizing the local concentration. Although all methods were significantly effective, the dosages also varied within studies, from 1 to 3 g of TXA in 10 to 100 mL of normal saline [9]. The minimum required concentration is also reported in the literature to be 10 to 20 mg/mL [39, 40]. Such information was the basis for the protocol of intraarticular injection of 1 g TXA per 50 mL normal saline in the present study [9, 39, 40].

The limitations of the present study must be noted. The present study is a retrospective study, which may be subject to selection bias. However, the treatment decision of whether to include intraarticular TXA in the surgical protocol was based on the date of the surgery, not on other parameters. In addition, propensity score matching was employed to match the patients not only with demographic details but also using the physiological status reflected by preoperative Hb and ASA-PS. Haemodynamic outcome parameters and the need for transfusion, which is strictly triggered by the postoperative nadir Hb, can be greatly influenced by the preoperative status. Hence, the propensity score matching of preoperative Hb and ASA-PS was believed to have strengthened the results of the present study, which showed dramatic contrasts between the two groups. Another limitation of this study is the lack of an experimental group treated with TXA without rivaroxaban and a control group not treated with either TXA or rivaroxaban. However, as postoperative chemical thromboprophylaxis after TKA is now a standard of care, such a study design may have raised ethical issues.

Despite these limitations, this study is among the first to successfully prove the effectiveness and safety of the combined use of topical, intraarticular TXA with rivaroxaban. Previous publications have reported the high potency of the individual regimens and the limitations when not used in conjunction [13, 14, 21]. Therefore, the present study can be of value in choosing the optimal perioperative management for TKA patients, effectively lowering blood loss, and reducing transfusions and wound complications while offering the best protection available against DVT.

## Conclusions

The combined use of topical, intraarticular TXA with rivaroxaban in patients undergoing TKA is a safe and effective approach to reduce blood loss, the need for transfusions and wound complications without elevating the risk of DVT.

## Abbreviations

ASA-PS: American Society of Anesthesiologists physical status
BMI: Body mass index
CI: Confidence interval
DVT: Deep vein thrombosis
Hb: Haemoglobin
LMWH: Low molecular weight heparin
POD: Postoperative day
RV: Rivaroxaban-only
RVTX: Rivaroxaban plus topical TXA
TKA: Total knee arthroplasty
TXA: Tranexamic acid
